# A checklist of Nigerian ants (Hymenoptera, Formicidae): a review, new records and exotic species

**DOI:** 10.3897/BDJ.12.e99555

**Published:** 2024-01-29

**Authors:** Bunmi Omowumi Jimoh, Kiko Gómez, Kehinde Abike Kemabonta, Winifred Ayinke Wakanjuola, Ethel Emmarantia Phiri, Palesa Natasha Mothapo

**Affiliations:** 1 University of Lagos, Lagos, Nigeria University of Lagos Lagos Nigeria; 2 Independent Researcher, Barcelona, Spain Independent Researcher Barcelona Spain; 3 Stellenbosch University, Stellenbosch, South Africa Stellenbosch University Stellenbosch South Africa

**Keywords:** Afrotropics, ant diversity, native and non-native ants, Lagos, West Africa

## Abstract

**Background:**

Ants are one of the most ubiquitous, widespread and abundant groups of animals on Earth. They are eusocial and are well noted for their important ecosystem services such as nutrient recycling, seed dispersal, engaging in mutualistic associations with other organisms, as well as serving as predators and scavengers. Although Africa has been recognised as a global hotspot for ant diversity, African ant genera are not as well-known when compared with other regions. The last checklist of Nigerian ants was compiled and published in the 1970s. To contribute to new knowledge on West African ant genera and Nigeria in particular, we conducted a review of the ant species of Nigeria using 132 scientific publications mostly compiled in the database www.antmaps.org, along with a survey of ant species of Lagos and Oyo States in Nigeria which was conducted between 2018 and 2020. The study aimed to ascertain the diversity of Nigerian ant genera, as well as to confirm the presence of previously recorded species and add new species to the current checklist of Nigerian ants, based on the 1970 survey.

**New information:**

As many as 106 species were recorded from the survey in the current study, of which 28 are new to Nigeria and additional 28 are identified to the morphospecies level. In total, 317 species from 10 subfamilies and 64 genera are now recorded from Nigeria, including 11 invasive ants, of which six are new to Nigeria. The following eleven species that were included in the 1970 checklist were excluded from the current list, mostly due to previous misidentifications: *Aenictusrotundatus* Mayr, 1901; *Anochetusjonesi* Arnold, 1926; *Camponotusbarbarossamicipsa* Wheeler, 1992; *Camponotusforaminosusdorsalis* Santschi, 1926; *Camponotusrufoglaucus* (Jerdon, 1851); *Cardiocondylazoserka* Bolton, 1982; *Messorbarbarus* (Linnaeus, 1767); *Odontomachushaematodus* (Linnaeus, 1758); *Technomyrmexalbipes* (Smith, 1861); *Tetramoriumdecem* Forel, 1913 and *Tetraponerapenzigi* (Mayr, 1907).

## Introduction

Ants (Hymenoptera, Formicidae) are very important in both natural and managed ecosystems because of their high diversity, abundance and interactions with fauna, flora and physical factors in their environment (Hölldobler and Wilson 1990, Subedi 2016). Their activities profoundly affect ecosystem dynamics through the modification, maintenance and creation of habitats for other organisms in the ecosystem (Jouquet et al. 2006, Frouz and Jílková 2008, Subedi 2016). Moreover, they have been used as bioindicators of land restoration success, land degradation and the conservation value of land parcels (King et al. 1998, Majer et al. 2007), specifically in relation to mine site restoration (Andersen 1997). They have evolved to become the most species rich and ecologically diverse social insects since their origin about 120 million years ago (Hölldobler and Wilson 1990, Grimaldi and Engel 2005). Despite records indicating an estimated 14,114 species, 346 genera and 16 subfamilies in the world (Bolton 2023), a very high number of species are yet to be discovered and officialy described (Hölldobler and Wilson 1990, Ward 2000). Thus, the diversity of ant species, both identified and unidentified, could far exceed twenty-five thousand (Wilson 2003, Bolton 2007).

Due to their social structure, ability to form large colonies, high reproductive rates, as well as the ability of their reproductive castes to fly long distances, ants can rapidly establish and dominate in new environments (both natural and disturbed) (Vonshak and Gordon 2015). Globally, many species of ants have rapidly spread to many regions outside their natural geographic distribution where they have established, spread and become invasive (Wetterer 2009a, Wetterer 2009b, Wetterer 2009c, Wetterer 2010a, Wetterer 2010b, Wetterer 2010c, Wetterer 2011a, Wetterer 2012a, Wetterer 2012b, Simberloff et al. 2013, Wauters et al. 2014, Wetterer 2019). These ants have become a global threat to biodiversity and general pests of agricultural and economic importance in all the regions where they have been introduced and become invasive (McGlynn 1999, Suarez et al. 2010, Pimentel 2011). Indeed, five ant species have made it on to the Global Invasive Species Database (GISD) of the 100 worst invasive species in the world: *Anoplolepisgracillipes* (Smith, 1857)- Yellow crazy ant (#6), *Linepithemahumile* (Mayr, 1868)- Argentine ant (#48), *Pheidolemegecephala* (Fabricius, 1793)- African big head ant (#68), *Solenopsisinvicta* Buren, 1972- Red Imported Fire Ant (#86) and *Wasmanniaauropunctata* (Roger, 1863)- Little fire ant (#100) (GISD 2023). Although research on invasive ants is abundant globally, there is a paucity of studies in the African context (Mothapo and Wossler 2017). Moreover, there is a dearth of information quantifying the presence and/or absence of invasive ant species on the continent. Recent work in Côte d’Ivoire traced invasive ants in urban settings and showed a record number of exotic and invasive species in urban, peri-urban and natural environments (Kouakou et al. 2018a, Kouakou et al. 2018b).

Africa, being mainly tropical, has been recognised as one of the hotspots for ant diversity (Ward 2000, Guénard et al. 2012). Within the tropical regions of Africa, especially West Africa, ant species richness appears to be comparatively lower than in other regions of similar latitude, but this may be due to lack of comprehensive sampling compared to other regions (Dunn et al. 2007, Guénard et al. 2012, Kouakou et al. 2018a, Kouakou et al. 2018b). Recent generic revisions seem to support the view that the Afrotropical fauna is widely underestimated (Gómez 2020). Since the year 2000, nine new genera have been described for the region and, in the 14 genera revised since 2000, the number of described species has increased from 92 to 214, a 133% increase (Gómez 2020).

Early ant-listing publications in Nigeria were published by Booker (1968), Eguagie (1971) and Adenuga (1975) as stated in Taylor (1977). In their studies of cocoa tree cultivars, Booker (1968) recorded 19 species and Eguagie (1971) recorded 27. The most comprehensive early ant collection in Nigeria was the one maintained in the Cocoa Research Institute of Nigeria (CRIN) Entomological Museum (158 species from 51 genera), mostly collected by Dr. Barry Bolton in 1969 (Taylor 1977). The first published comprehensive ant survey was carried out in 1976, where Dr. Brian Taylor collected ants from the Gambari Experimental Station of the Cocoa Research Institute of Nigeria in Ibadan and other cocoa growing areas of western Nigeria (Taylor 1976). The main purpose of this survey, which recorded 130 species, was to study the role of ants in the spread of black pod disease of cocoa (Taylor 1976, Taylor 1977, Taylor 1978, Taylor and Adedoyin 1978, Taylor 1979, Taylor 1980a, Taylor 1980b). Apart from the work done in the 1970s by Barry Bolton and Brian Taylor, no comprehensive published studies exist and there is a need for update of the checklists. Moreover, there have been no records of invasive ant species in Nigeria. Therefore, this study was carried out to ascertain the presence of invasive ants in Nigeria and to reassess the list of ant species in the current checklist.

## Materials and methods

### Sampling locations

**Figure 1.**
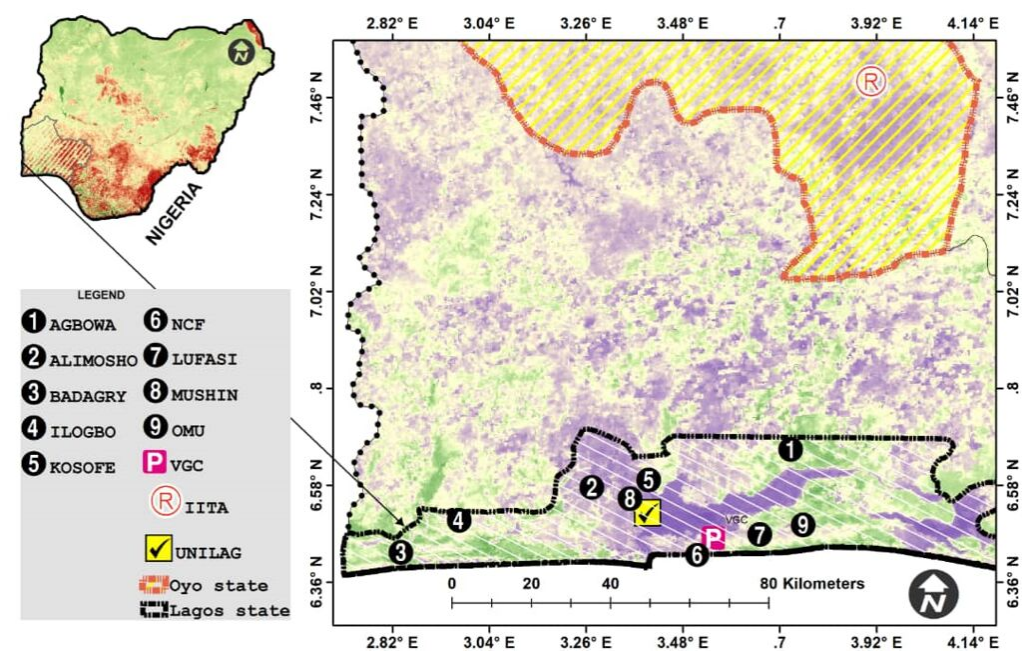
Map of Lagos State and part of Oyo State in Nigeria showing the locations where ants were sampled for the current survey. Specific localities are indicated in Table 2.

The study locations are in Lagos and Oyo States, Nigeria. A large part of the sampling was conducted in Lagos State (Fig. 1 and Table 2), but an additional collection was made in the International Institute of Tropical Agriculture (IITA) Ibadan, Oyo State. The sampling locations in Lagos were: Lekki Conservation Center (LCC/NCF), Lekki Urban Forestry and Animal Shelter Initiatives (LUFASI), Omu Resort, Agbowa, Badagry, Ilogbo Eremi, Ilupeju in Mushin Local Government, Ketu in Kosofe Local Government, Ikotun in Alimosho Local Government, Victoria Garden City (VGC) and University of Lagos (UNILAG).

### Ant sampling and identification

Ants sampling was done from 2018 to 2020, using three different sampling methods: pitfall traps, baiting (sardine, peanut butter and jam) and direct sampling (hand collection). Ants were collected from urban, agroecosystem (cassava plantation) and semi-natural areas (secondary forest). The samples were stored inside vials containing 75% alcohol. All collected ants were cleaned, sorted and identified initially to the genus level using a stereomicroscope and keys in Bolton (1994) and Fisher and Bolton (2016). Further identification to species level was conducted with keys available on Antwiki using the original scientific journals cited there. Identified material was compared with type images at Antweb (www.antweb.org) when available and distributions checked with Antmaps (www.antmaps.org). Antmaps is the largest database of ant species, consisting of 1.72 million records extracted from over 8811 publications and 25 existing databases which are updated on a weekly basis (Guénard et al. 2017). Antweb is the main image database including photographs of type specimens for most of the described ant species, but also used to store records, natural and taxonomic history of every ant species. One hundred and thirty two scientific journals from these repositories were reviewed in order to establish the existing ant species in Nigeria. Voucher specimens are available in the Kiko Gómez Collection (KGAC) in Spain. All available information has been uploaded into the Antweb database for public access.

We have identified four species to a subspecies status *Pheidolecaffrasenilifrons* Wheeler, 1922, *Lepisiotacapensislaevis* (Santschi, 1913), *Crematogasterkneripronotalis* Santschi, 1914 and *Pheidoleexcellensweissi* Santschi, 1910. We do not feel comfortable with the subgeneric names, but the current state of the taxonomy in these genera makes it impossible for us to judge the validity of these names. Identification has been done by comparing the material with available types in Antweb and, although adopting these names suggests that we agree with its validity as a good species, we prefer to wait until a thorough revision for the group is published. We recognise morphological differences between the types of the names we have adopted and the other subspecies, but they could be due to a number of reasons (regional or individual variation, for instance), so we refrain from modifying its status with the information we have gathered.

Below is the checklist of Nigerian ants. All the species collected in the present survey are denoted by asterisks, the ones with double asterisks (**) being new records for Nigeria.

## Checklists

### A checklist of Nigerian ants

#### Amblyoponinae

D4378037-59CD-5B9A-B76F-9E7CFA85ACF8

#### Xymmer muticus

(Santschi, 1914)

7AC10652-CA2B-57BB-9DA5-940E7A15BF92

##### Notes

(Santschi 1914, Wheeler 1922, Lévieux 1972, Gotwald and Lévieux 1972, Brown 1960, Medler 1980, Yoshimura and Fisher 2012) .

#### Apomyrminae

495C951E-453F-5CD3-B262-7861C33A7543

#### Apomyrma stygia

Brown et al., 1971

2187C5FD-2E9D-5C1C-ABAC-60E70D2A5A29

##### Notes

(Medler 1980)

#### Dolichoderinae

642C8BFB-3258-5B88-ACCD-B6F5C64438FA

#### Axinidris denticulata

(Wheeler, 1922)

13B56ECB-092A-53F8-AC37-8839328D363A

##### Notes

(Taylor 1978)

#### Axinidris kinoin

Shattuck, 1991

4E7861B3-6995-5E37-9671-D730932DE0C1

##### Notes

(Shattuck 1991, Shattuck 1994, Snelling 2007)

#### * Tapinoma carininotum

Weber, 1943

EC48447C-0B84-5BD7-95D8-039B437C4444

##### Notes

(Taylor et al. 2018)

New Records: 1

#### Tapinoma lugubre

Santschi, 1917

F11B4303-2DA1-5031-A96E-F38CD9E92EA8

##### Notes

(Braet and Taylor 2008)

#### Tapinoma melanocephalum

(Fabricius, 1793)

9DBBEB18-423B-526A-9522-E2C9214DCAFA

##### Notes

(Taylor 1978, Medler 1980, Wetterer 2009b)

#### Technomyrmex andrei

Emery, 1899

44CCD597-CAEB-5122-862C-9003F908968D

##### Notes

(Medler 1980)

#### Technomyrmex moerens

Santschi, 1913

5023EBC6-FB74-5B6E-9FC8-2A0FF4A66D89

##### Notes

(Bolton 2007)

#### Technomyrmex nigriventris

Forel, 1910

32E29D9E-E5C7-5F04-88B6-ABDA80BFFDC4

##### Notes

(Bolton 2007)

#### Technomyrmex parviflavus

Bolton, 2007

7FEBEB76-8F82-5F05-86F3-56250757E750

##### Notes

(Bolton 2007)

#### Technomyrmex semiruber

Emery, 1899

EF54C3D0-ABEF-5752-9A92-67ED4D2B8EA2

##### Notes

(Braet and Taylor 2008)

#### Dorylinae

7F86B7A3-15B1-575B-8D7C-EC018A58BA1E

#### Aenictus decolor

(Mayr, 1879)

302A9926-D20C-557D-9B77-CF0C53DA6DED

##### Notes

(Wheeler 1922, Baroni Urbani 1977a, Gotwald and Leroux 1980, Medler 1980)

#### Aenictus guineensis

Santschi, 1924

15685767-8271-53C8-820E-575325F931BD

##### Notes

(Medler 1980, Taylor 1980a, Taylor et al. 2018)

#### Aenictus vagans

Santschi, 1924

0447EA25-8FFC-5A7A-94F6-4DD6DBD0B283

##### Notes

(Baroni Urbani 1977a)

#### Dorylus affinis

Shuckard, 1840

9CFE4219-E318-521E-A2AE-F1FBE913C5F6

##### Notes

(Medler 1980, Ewuim 2007)

#### Dorylus atratus

Shuckard, 1840

A525EC42-2ADD-58EC-B570-46D86C698B7C

##### Notes

(Emery 1895, Wheeler 1922, Medler 1980, Borowiec 2016)

#### ** Dorylus braunsi

Emery, 1895

F1B40264-4C28-5B2A-8608-2612EAA7A504

##### Notes

New record for Nigeria

New Records: 2, 9

#### Dorylus depilis

Emery, 1895

C3291A32-B18A-57E9-BE20-1423713D4DBC

##### Notes

(Wheeler 1922, Medler 1980)

#### Dorylus emeryi

Mayr, 1896

68FBBA89-3F39-5E54-AA40-7F8F6782689C

##### Notes

(Medler 1980)

#### Dorylus fimbriatus

(Shuckard, 1840)

03A21D6C-DCF6-5CE3-A1A0-BCF7272E7948

##### Notes

(Medler 1980, Taylor 1980a)

#### Dorylus fulvus

(Westwood, 1839)

88E077F5-7EB2-56DE-AF7E-947B5EB2181C

##### Notes

(Medler 1980)

#### Dorylus gribodoi

Emery, 1892

4764DEB9-F7D5-5621-A9E5-1FA1ABFE6260

##### Notes

(Wheeler 1922, Medler 1980, Schöning et al. 2008)

#### Dorylus helvolus

(Linnaeus, 1764)

35BAE0A5-BF79-5FFE-9ACA-2356F2A35793

##### Notes

(Wheeler 1922, Medler 1980, Taylor 1980a, Taylor et al. 2018)

#### Dorylus kohli

Wasmann, 1904

E8375738-8A50-564F-B30F-D7E1DFA32EF2

##### Notes

(Medler 1980, Taylor 1980a, Taylor et al. 2018)

#### Dorylus nigricans

Wasmann, 1904

20332F13-319E-5911-9F18-08A86786F541

##### Notes

(Emery 1910, Wheeler 1922, Taylor 1980a, Medler 1980, Nwosu and Lawal 2010, Ewuim et al. 2011)

#### Dorylus nigricans burmeisteri

(Shuckard, 1840)

B10E9B1C-D65C-5746-8F5C-C168AACC2AE9

##### Notes

(Santschi 1914, Wheeler 1922)

#### Dorylus nigricans rubellus

(Savage, 1849)

BACE355A-7011-51C3-B023-518E7A34201F

##### Notes

(Schöning and Moffett 2007)

#### Dorylus rufescens

Santschi, 1915

61A092FD-B77E-54A0-8C7C-1B8C1754DC1F

##### Notes

(Menozzi 1926)

#### Dorylus savagei

Emery, 1895

8A69EF21-6CE7-5D3C-A664-8034BF29C4EB

##### Notes

(Medler 1980)

#### Dorylus savagei mucronatus

Emery, 1899

0F4CB13B-CFC0-5C86-B2B1-A839D20802B5

##### Notes

(Emery 1899, Forel 1901, Emery 1910, Wheeler 1922, Borowiec 2016)

#### Dorylus spininodis

Emery, 1901

6EB206F0-2CC9-5D02-B592-AEDBF3FF66AF

##### Notes

(Santschi 1914, Wheeler 1922, Medler 1980)

#### Lioponera foreli

(Santschi, 1914)

B3155DEC-22F3-5E74-87CF-A75F207C0451

##### Notes

(Taylor 1976, Medler 1980)

#### Parasyscia cribrinodis

Emery, 1899

34FA4F32-DA0C-578E-87C4-B3DC796F73B6

##### Notes

(Taylor 1976)

#### Parasyscia sudanensis

(Weber, 1942)

BA0F9484-9CA9-5EC8-9313-B183E1A19404

##### Notes

(Brown 1975)

#### Simopone conradti

Emery, 1899

05BED52B-583D-58F4-9BE9-88C1F316D0DB

##### Notes

(Bolton and Fisher 2012)

#### Zasphinctus rufiventris

(Santschi, 1915)

C64AB996-3BB3-593C-8414-30B529350231

##### Notes

(Medler 1980)

#### Formicinae

9F43E579-4F07-57D9-9D74-D90A80CAEEF7

#### Camponotus aberrans

Mayr, 1895

501B4182-C4B5-5709-ADC9-36CFCF68E5CC

##### Notes

(Taylor 1978)

#### * Camponotus acvapimensis

Mayr, 1862

803F9C19-143C-5816-A7D8-9FBE37C40174

##### Notes

(Forel 1910, Forel 1913, Wheeler 1922, Menozzi 1926, Brown 1956, Taylor 1978, Taylor and Adedoyin 1978, Medler 1980, Ewuim 2007, Ewuim et al. 2011)

New Records: 1, 2, 3, 5, 7, 8, 10

#### Camponotus barbarossa micipsa

Emery, 1920

6DE9AF6A-2BAF-59CE-A218-7D2C56138932

##### Notes

(Taylor 1978)

#### Camponotus bayeri

Forel, 1913

7BC92EC9-FE8C-515E-9EE8-64D26F4CF041

##### Notes

(Weber 1964)

#### Camponotus bituberculatus

André, 1889

7E1E133F-ABE5-5284-A8D0-AC00A0AFC292

##### Notes

(Santschi 1915)

#### Camponotus brutus

Forel, 1886

BF4E8B9B-EA43-5762-B58D-6D2893B2A300

##### Notes

(Taylor 1978, Medler 1980)

#### Camponotus chrysurus

Gerstäcker, 1871

772AB987-F3D7-5F8E-9DF6-B24EA3B33658

##### Notes

(Medler 1980)

#### Camponotus compressiscapus

André, 1889

AEBBB320-B252-5670-9360-42F886575F59

##### Notes

(Santschi 1915)

#### Camponotus congolensis

Emery, 1899

85141EED-4F0F-573F-A902-BFBCB020A202

##### Notes

(Bernard 1953)

#### * Camponotus flavomarginatus

Mayr, 1862

55236BD4-C1BA-5905-A83C-381A6817309D

##### Notes

(Taylor 1978, Medler 1980)

New Records: 4, 5, 10

#### Camponotus foraminosus

Forel, 1879

A65DAE76-C27F-5AF7-AC26-D43236F7CB25

##### Notes

(Santschi 1915, Wheeler 1922, Medler 1980)

#### Camponotus foraminosus deductus

Santschi, 1915

B36A42BF-B4E1-539E-80CC-C620BB388E96

##### Notes

(Santschi 1915 as s. str.; Bolton 1975b, Taylor 1978, Taylor and Adedoyin 1978, Medler 1980 as ssp. dorsalis)

#### * Camponotus haereticus

Santschi, 1914

EA0C2DBE-36D4-5AC6-B79D-5ACD45D34300

##### Notes

(Santschi 1914, Santschi 1915, Wheeler 1922)

New Records: 1, 6, 8, 10

#### * Camponotus maculatus

(Fabricius, 1782)

FEE4436D-A43F-5DA8-98E9-0E2287378281

##### Notes

(Santschi 1914, Santschi 1915, Wheeler 1922, Prins 1963, Bolton 1969, Taylor 1978, Medler 1980, Ewuim 2007, Ewuim et al. 2011)

New Records: 2, 3, 5, 6, 8, 9, 10, 11

#### Camponotus perrisii

Forel, 1886

16F69239-850C-5107-A0FC-07C42E3E4C0E

##### Notes

(Medler 1980, Ewuim 2007)

#### Camponotus perrisii nigeriensis

Santschi, 1914

BEF67F3D-C082-5937-BC0C-0EBCC02E6D69

##### Notes

(Santschi 1914, Wheeler 1922)

#### ** Camponotus schoutedeni

Forel, 1911

3396DAB4-9C56-5BB6-AF7D-8EFE17C89E54

##### Notes

New record for Nigeria

New Records: 1, 2, 10

#### * Camponotus sericeus

(Fabricius, 1798)

44AE31AB-BAC1-5051-A098-0422C697C881

##### Notes

(Wheeler 1922, Prins 1963, Prins 1964, Medler 1980)

New Records: 6, 10

#### Camponotus solon

Forel, 1886

40D07FFE-1D0E-565A-B377-804D29314719

##### Notes

(Medler 1980)

#### Camponotus solon chiton

Emery, 1925

3DC12525-340A-5B94-8B6B-2604752BDC33

##### Notes

(Wheeler 1922)

#### Camponotus vestitus

(Smith, 1858)

7EC35CC0-A954-5242-8788-1DD108882B97

##### Notes

(Medler 1980)

#### Camponotus vestitus comptus

Santschi, 1926

C7843405-A694-5A39-9005-C2EC50A41166

##### Notes

(Santschi 1937a)

#### * Camponotus vividus

(Smith, 1858)

767C9FDC-2E19-5733-A3EE-47FA2057B480

##### Notes

(Wheeler 1922, Taylor 1978, Taylor and Adedoyin 1978, Medler 1980)

New Records: 4, 5, 6, 8, 10

#### Camponotus vividus meinerti

Forel, 1886

3B44321F-9DB5-5AF7-85BF-9DE31CE38813

##### Notes

(Santschi 1914)

#### Cataglyphis bicolor

(Fabricius, 1793)

89570DA1-D90F-597F-9453-BACD42C5903E

##### Notes

(Medler 1980)

#### Cataglyphis bicolor seticornis

(Emery, 1906)

C6843BE6-5C64-5224-B311-72998D0223BC

##### Notes

(Medler 1980)

#### ** Lepisiota ambigua

(Santschi, 1935)

B53E257C-B79F-578B-9ADA-6358C068CAD4

##### Notes

New record for Nigeria

New Records: 6

#### Lepisiota cacozela

(Stitz, 1916)

2FA66A79-A922-514D-92E5-DBD9DEC574C8

##### Notes

(Santschi 1914, Wheeler 1922)

#### * Lepisiota canescens

(Emery, 1897)

50062EE6-1180-5CF1-B167-23BBE8834C3D

##### Notes

(Santschi 1914, Wheeler 1922)

New Records: 1, 2, 4, 5, 6, 8, 10

#### Lepisiota capensis

(Mayr, 1862)

BC9E5D66-0085-5F1D-A8AE-6DE247E9235C

##### Notes

(Taylor 1978, Taylor and Adedoyin 1978, Medler 1980)

#### Lepisiota capensis guineensis

(Mayr, 1902)

D134BAF1-4203-59BD-8526-E34312408AD6

##### Notes

(Taylor et al. 2018)

#### ** Lepisiota capensis laevis

(Santschi, 1913)

5766287D-C81B-5D2A-9ECA-B93168C2F323

##### Notes

New record for Nigeria:

New Records: 1, 2, 6, 8, 9, 10, 11

#### Lepisiota incisa

(Forel, 1913)

3E41045F-F6DA-5B8E-9CA6-29382F600F24

##### Notes

(Medler 1980)

#### Lepisiota monardi

(Santschi, 1930)

77D11B29-E05C-55A7-A0E0-2DC254D528D2

##### Notes

(Taylor et al. 2016)

#### Lepisiota spinosior

(Santschi, 1930)

4CDA146F-0037-511D-8624-68199805D986

##### Notes

(Taylor 1978, Medler 1980)

#### Lepisiota validiuscula

(Emery, 1897)

1C751732-132D-58AA-A534-F756AE34D42D

##### Notes

(Taylor et al. 2018)

#### Nylanderia boltoni

LaPolla et al., 2011

B6166C0F-3359-5322-A051-1219BB629C31

##### Notes

(LaPolla et al. 2011)

#### ** Nylanderia bourbonica

(Forel, 1886)

16FA2D78-3FA4-5292-A9FA-9AA157A4E4AF

##### Notes

New record for Nigeria.

New Records: 2, 4, 8, 9, 11

#### Nylanderia lepida

(Santschi, 1915)

36CFF770-7F98-547D-9E4B-C8A6C1736344

##### Notes

(LaPolla et al. 2011)

#### Nylanderia scintilla

LaPolla et al., 2011

AA972D05-7993-53C8-9D90-B1696CD7C9ED

##### Notes

(LaPolla et al. 2011)

#### ** Nylanderia umbella

LaPolla et al., 2011

A534C4CD-82BA-56CB-9F53-6104CA6C192A

##### Notes

New record for Nigeria.

New Records: 1, 2, 8, 9, 10, 11, 12

#### * Oecophylla longinoda

(Latreille, 1802)

32C25A32-7BB8-56FF-A0C9-0DE3E6FDAD90

##### Notes

(Wheeler 1922, Menozzi 1926, Taylor 1978, Taylor and Adedoyin 1978, Medler 1980, Ewuim 2007, Badejo et al. 2011, Ewuim et al. 2011, Wetterer 2017a, Wetterer 2017b)

New Records: 1, 2, 4, 5, 8, 11

#### Oecophylla longinoda fusca

Emery, 1899

453E61FA-7DA2-519F-A3B3-74282B42FA75

##### Notes

(Wheeler 1922, Menozzi 1926, Cole and Jones 1948)

#### Paraparatrechina albipes

(Emery, 1899)

472FFA59-8707-5125-974C-BED154F21CFC

##### Notes

(LaPolla et al. 2010)

#### Paraparatrechina gnoma

LaPolla & Cheng, 2010

26E16FFB-A08E-5439-BAE3-06DAFC347841

##### Notes

(LaPolla et al. 2010)

#### Paraparatrechina subtilis

(Santschi, 1920)

FAB07AAC-540D-5E99-9418-71AC448F0993

##### Notes

(Braet and Taylor 2008)

#### * Paratrechina longicornis

(Latreille, 1802)

1A70EED7-6BF7-518B-BA4C-8C12D36D3200

##### Notes

(Santschi 1914, Wheeler 1922, Medler 1980, Wetterer 2008)

New Records: 1, 2, 4,, 5, 7, 8, 9, 10, 11, 12

#### Plagiolepis alluaudi

Emery, 1894

AAA14892-A89C-58FA-9150-D320BBBE1D8E

##### Notes

(Wetterer 2013b)

#### Plagiolepis brunni

Mayr, 1895

E71893E7-5949-5485-A310-81307407192C

##### Notes

(Taylor 1978, Medler 1980)

#### Polyrhachis concava

André, 1889

53FEB92F-59A6-5D70-ABC1-6598F577FE1B

##### Notes

(Taylor 1978)

#### Polyrhachis decemdentata

André, 1889

0155EC16-0A13-53F4-8EB4-232B3359F539

##### Notes

(Bolton 1973b, Taylor 1978, Medler 1980)

#### Polyrhachis fissa

Mayr, 1902

99176C53-812F-59F8-8F1C-93EDFACB3568

##### Notes

(Medler 1980)

#### Polyrhachis laboriosa

Smith, 1858

3AEC699F-A3A7-5227-9A81-014C9A821126

##### Notes

(Santschi 1914, Wheeler 1922, Bolton 1973b, Taylor 1978, Medler 1980)

#### * Polyrhachis militaris

(Fabricius, 1782)

F5559821-B009-5CE6-A4B8-52E0C032CBAD

##### Notes

(Forel 1901, Wheeler 1922, Bernard 1953, Bolton 1973b, Taylor 1978, Medler 1980, Rigato 2016)

New Records: 8

#### Polyrhachis monista

Santschi, 1910

AE8B309F-0800-54EB-977F-913FD641374E

##### Notes

(Santschi 1914, Bolton 1973b, Taylor 1978, Medler 1980)

#### Polyrhachis otleti

Forel, 1916

5AD99D7C-9A4B-574C-BB6C-FF52B2824F27

##### Notes

(Bolton 1973b, Taylor 1978, Medler 1980)

#### Polyrhachis phidias

Forel, 1910

304A8210-2005-5C29-924B-F7C5F08C245A

##### Notes

(Taylor 1978)

#### Polyrhachis rufipalpis

Santschi, 1910

3CD99395-23D3-5BEA-B415-32399F17128A

##### Notes

(Taylor 1978)

#### Polyrhachis schistacea

(Gerstäcker, 1859)

DE3071CB-1DC0-5E69-BDCA-95ECC20FD760

##### Notes

(Bolton 1973b, Medler 1980)

#### Polyrhachis viscosa

Smith, 1858

13AD4E0A-2022-5F0C-A7B1-55AFA253C0B8

##### Notes

(Bolton 1973b, Medler 1980)

#### Polyrhachis weissi

Santschi, 1910

A4AEB1B1-2C6A-5005-9B36-E739550E2585

##### Notes

(Taylor 1978)

#### ** Tapinolepis pernix

(Viehmeyer, 1923)

F9EF2D65-DB8D-59AD-B45E-48B5D399963E

##### Notes

New record for Nigeria

New Records: 2

#### Leptanillinae

2A5E0840-00CC-5B3B-ADCA-30DB593874AF

#### Leptanilla africana

Baroni Urbani, 1977

92535037-A6E3-54B3-BC1B-19F71AD7CCB1

##### Notes

(Zolessi and Abenante 1973, Baroni Urbani 1977b, Boudinot 2015)

#### Myrmicinae

C5C8EDF4-8065-53E7-A06D-29E256506F56

#### * Atopomyrmex cryptoceroides

Emery, 1892

8EA2B75B-6754-5BCC-8F33-5C8377802169

##### Notes

(Santschi 1914, Wheeler 1922)

New Records: 5, 6

#### Atopomyrmex mocquerysi

André, 1889

99B7712D-11F3-5BD9-A34E-17278D3F21CB

##### Notes

(Taylor 1980a, Medler 1980, Bolton 1981a)

#### Baracidris meketra

Bolton, 1981

5B662F70-FC13-5FC1-8B58-DF55D18045D0

##### Notes

(Bolton 1981a, Fernández 2003)

#### Bondroitia lujae

(Forel, 1909)

58D4B12A-6777-5557-BD54-C6EBE01E90EC

##### Notes

(Taylor 1980a)

#### Calyptomyrmex barak

Bolton, 1981

81494AB5-2FEC-51B1-BCD0-2013B00829B7

##### Notes

(Bolton 1981b)

#### Calyptomyrmex nummuliticus

Santschi, 1914

8BBDAF11-D95E-568E-A54C-7966947EA98E

##### Notes

(Bolton 1981b)

#### * Cardiocondyla emeryi

Forel, 1881

0F478351-5742-51DB-9C59-BA3017CACEB6

##### Notes

(Taylor 1979, Medler 1980, Bolton 1982, Kugler 1984, Seifert 2003, Wetterer 2012a, Mohammadi et al. 2012)

New Records: 1, 2, 3, 4, 8, 10

#### Cardiocondyla neferka

Bolton, 1982

D08CF539-1E29-558C-B6B0-BE67CDAB3E19

##### Notes

(Seifert 2003)

#### ** Cardiocondyla sekhemka

Bolton, 1982

160558EB-29DC-5587-AABB-7DAD60BBD84D

##### Notes

New record for Nigeria

New Records: 2

#### * Cardiocondyla shuckardi

Forel, 1891

3A77C766-46F4-5168-89B7-8346B2423AA4

##### Notes

(Bolton 1982)

New Records: 1, 2, 6, 8, 10, 11

#### ** Cardiocondyla weserka

Bolton, 1982

5F83BB5D-8445-571E-90C8-7FD8CC1B8B6C

##### Notes

New record for Nigeria

New Records: 2

#### * Cardiocondyla yoruba

Rigato, 2002

2DAE8762-CBF5-5425-A46A-C7E5DF789EA1

##### Notes

(Seifert 2003)

New Records: 1, 2, 3, 6, 7, 9, 12

#### Cardiocondyla zoserka

Bolton, 1982

53D069E5-87DB-59AE-8D59-2DF128D82B68

##### Notes

(Bolton 1982, Heinze 2019)

#### Carebara distincta

(Bolton & Belshaw, 1993)

C5EB2E65-7C8F-5BA6-99F9-0066031E44BA

##### Notes

(Bolton and Belshaw 1993)

#### Carebara silvestrii

(Santschi, 1914)

81DD6667-BB6A-50FE-9C1D-B708C23CA860

##### Notes

(Medler 1980, Taylor 1980a)

#### Carebara termitolestes

(Wheeler, 1918)

5B15B977-F6EB-5BFC-898A-BE970673C14F

##### Notes

(Bolton 1969, Taylor 1980a)

#### Cataulacus bequaerti

Forel, 1913

8B61E1CE-B8D4-5E04-83DE-851A4B7D6C02

##### Notes

(Bolton 1974a)

#### Cataulacus boltoni

Snelling, 1979

FCF1C7FF-0DA6-5EAC-A655-A3CFCE856D5B

##### Notes

(Snelling 1979, Bolton 1982)

#### Cataulacus brevisetosus

Forel, 1901

41BD4C0A-BF48-5B07-9FC3-97518ED2BEC4

##### Notes

(Bolton 1974a, Taylor 1979, Medler 1980)

#### Cataulacus difficilis

Santschi, 1916

E8619F81-124D-5CA9-850C-39241861C0F1

##### Notes

(Taylor 1979)

#### Cataulacus egenus

Santschi, 1911

E9B42344-FD35-55C6-8F06-1F0482B0E3C1

##### Notes

(Bolton 1974a, Taylor 1979, Medler 1980, Bolton 1982)

#### * Cataulacus guineensis

Smith, 1853

7EC43CB6-CC2E-5249-ABE5-26F73C37DB28

##### Notes

Santschi 1914, Wheeler 1922, Bolton 1974a, Taylor and Adedoyin 1978, Taylor 1979, Medler 1980, Bolton 1982)

New Records: 10

#### Cataulacus huberi

André, 1890

40E31E46-B486-5B67-8691-BB0BA27CBFE6

##### Notes

(Bolton 1974a, Medler 1980, Bolton 1982)

#### * Cataulacus lujae

Forel, 1911

E6BCCF6B-E076-5971-92B7-67BC248BD5D9

##### Notes

(Bolton 1982)

New Records: 1

#### Cataulacus mocquerysi

André, 1889

316F80F4-0331-5E43-8434-CCB9551FF8DD

##### Notes

(Bolton 1974a, Taylor 1979, Medler 1980, Bolton 1982)

#### Cataulacus moloch

Bolton, 1982

A83138D9-FC5F-5B59-925C-18F0B821754E

##### Notes

(Bolton 1982)

#### Cataulacus pullus

Santschi, 1910

C8C4B961-3A05-58B1-8837-101B9336E1B0

##### Notes

(Bernard 1953)

#### Cataulacus pygmaeus

André, 1890

053175BD-C058-5CEA-A7A7-32EE552044B0

##### Notes

(Taylor 1979, Medler 1980)

#### Cataulacus taylori

Bolton, 1982

E9E0A522-44A6-56FB-8A05-F30E7557C27D

##### Notes

(Bolton 1982)

#### Cataulacus traegaordhi

Santschi, 1914

C01BA19D-3802-52B2-80ED-C43ED09570C6

##### Notes

(Bolton 1982)

#### Cataulacus vorticus

Bolton, 1974

F057C5F3-D0A7-5F59-8903-753E9168E4F5

##### Notes

(Bolton 1974a, Taylor 1979, Medler 1980, Bolton 1982)

#### * Cataulacus weissi

Santschi, 1913

820E60FC-001B-524D-88A8-078842D86829

##### Notes

(Taylor 1979)

New Records: 1

#### Crematogaster africana

Mayr, 1895

82234523-32C7-540A-87E5-1D9EE98772C6

##### Notes

(Forel 1910, Wheeler 1922, Soulié and Dicko 1965, Adenuga 1975, Taylor and Adedoyin 1978, Taylor 1979, Medler 1980, Nwosu and Lawal 2010)

#### Crematogaster africana alligatrix

Forel, 1911

DB6B48BB-04FF-5D13-82D6-44B8B66C78F9

##### Notes

(Forel 1913, Wheeler 1922, Soulié and Dicko 1965)

#### Crematogaster batesi

Forel, 1911

768CB77B-E785-5BF3-BE14-6D0C8577C224

##### Notes

(Wheeler 1922, Soulié and Dicko 1965)

#### Crematogaster bequaerti

Forel, 1913

D3D18780-8B92-5F2F-BB1D-2C22D8B3156E

##### Notes

(Taylor and Adedoyin 1978, Taylor 1979, Medler 1980)

#### Crematogaster brunneipennis

André, 1890

00DFEC91-8280-5740-BA4B-44A6E3F512C2

##### Notes

(Santschi 1933, Taylor et al. 2018)

#### Crematogaster brunneipennis yorubosa

Santschi, 1933

ADC4D560-9E18-56FE-933D-C33D65120117

##### Notes

(Santschi 1933)

#### Crematogaster buchneri

Forel, 1894

D132FBA0-573F-5836-8DCC-ED8CB2C929AB

##### Notes

(Menozzi 1926, Taylor 1979, Medler 1980)

#### Crematogaster buchneri graeteri

Forel, 1916

9E2CDD1E-9B91-5782-9C30-D349D805A9F2

##### Notes

(Forel 1913)

#### Crematogaster castanea

Smith, 1858

355731B9-81BD-537F-8863-9813743A412C

##### Notes

(Medler 1980)

#### Crematogaster clariventris

Mayr, 1895

206D8A6E-92D5-5142-9A16-5FF22ED1E646

##### Notes

(Forel 1913, Wheeler 1922, Soulié and Dicko 1965, Taylor and Adedoyin 1978, Taylor 1979, Medler 1980)

#### * Crematogaster depressa

Latreille, 1802

DFF00289-9082-591E-A306-F47A54D69D56

##### Notes

(Santschi 1914, Wheeler 1922, Soulié and Dicko 1965, Taylor 1979, Medler 1980)

New Records: 4. 10, 11

#### Crematogaster excisa

Mayr, 1895

A8F35475-EFF6-5DF6-A0F0-60849C0E7B51

##### Notes

(Taylor et al. 2018)

#### Crematogaster gabonensis

Emery, 1899

28180945-B711-5571-A8D6-63B87E2255AA

##### Notes

(Taylor and Adedoyin 1978, Taylor 1979, Medler 1980, Andersen 1997)

#### * Crematogaster gambiensis

André, 1889

CE31950D-38FF-53B7-807B-2218CA2DF9DB

##### Notes

(Taylor 1979, Medler 1980)

New Records: 4, 5, 12

#### Crematogaster gerstaeckeri

Dalla Torre, 1892

CB83C74F-2AD3-51CF-BCC0-6153BABA305F

##### Notes

(Taylor 1979, Medler 1980)

#### Crematogaster kneri

Mayr, 1862

B2BF182B-6CEE-5C64-AA40-CA70BF399CC0

##### Notes

(Taylor 1979, Medler 1980)

#### * Crematogaster kneri pronotalis

Santschi, 1914

4FA883C3-CFDE-5244-BFA9-EE967BEBC6AE

##### Notes

Santschi 1914, Wheeler 1922, Soulié and Dicko 1965)

New Records: 1, 2

#### Crematogaster kohli winkleri

Forel, 1909

984D6F0A-812B-562E-B5E4-CBE2E950A3CD

##### Notes

(Forel 1913, Wheeler 1922, Soulié and Dicko 1965)

#### ** Crematogaster lamottei

Bernard, 1953

0FFCB1CB-FC39-5305-AAD4-53560B174F3F

##### Notes

New record for Nigeria

New Records: 5

#### Crematogaster laurenti

Forel, 1909

CEB1E3D2-3681-5851-BF86-100F911FE26D

##### Notes

(Wheeler 1922, Soulié and Dicko 1965)

#### Crematogaster luctans

Forel, 1907

01A704DA-BB30-5C3F-B332-4E673464D4E2

##### Notes

(Adenuga 1975)

#### Crematogaster nigeriensis

Santschi, 1914

EA6F7BE0-C057-5C1B-A3C4-AF246C900554

##### Notes

(Santschi 1914, Wheeler 1922, Soulié and Dicko 1965, Medler 1980, Taylor et al. 2018)

#### Crematogaster solenopsides costeboriensis

Santschi, 1919

57F6D4DC-5FAA-5E41-A70F-B363F39407FE

##### Notes

(Taylor et al. 2018)

#### Crematogaster stadelmanni dolichocephala

Santschi, 1911

F26457C6-7B15-5B91-B2C6-4EA5430F2021

##### Notes

(Santschi 1914, Wheeler 1922, Soulié and Dicko 1965)

#### Crematogaster stigmata

Santschi, 1914

CD030224-3EC4-571C-912B-30EC1BD709DB

##### Notes

(Santschi 1914, Wheeler 1922, Soulié and Dicko 1965)

#### * Crematogaster striatula

Emery, 1892

78CF2038-06DD-59A3-B0AC-5AD867462C54

##### Notes

(Adenuga 1975, Taylor 1979, Medler 1980)

New Records: 8

#### Crematogaster wellmani

Forel, 1909

3F84AC76-9E2C-5F5F-986D-9645CDDAC626

##### Notes

(Forel 1910, Wheeler 1922, Soulié and Dicko 1965, Adenuga 1975, Taylor 1979, Medler 1980)

#### Crematogaster zavattarii

Menozzi, 1926

1820481B-9B49-5D31-9B71-9B73BEBE8E14

##### Notes

(Menozzi 1926, Taylor et al. 2018)

#### Dicroaspis cryptocera

Emery, 1908

C148C378-5465-57FA-A190-44CC5D50DE77

##### Notes

(Taylor 1979, Medler 1980)

#### Melissotarsus beccarii

Emery, 1877

3CB8F805-D297-5A0A-AF9E-51F37ECABA93

##### Notes

(Medler 1980)

#### Meranoplus inermis

Emery, 1895

5CDED494-A341-58FB-BDA4-ED51ED99D864

##### Notes

(Bolton 1981b)

#### Meranoplus nanus

André, 1892

9A906D85-C449-5BF9-A0B1-FDDDB219C931

##### Notes

(Taylor 1979, Medler 1980)

#### Messor galla

(Mayr, 1904)

9C9EEBC2-5B54-5863-84CD-749BA549BCFC

##### Notes

(Medler 1980, Bolton 1982)

#### Messor regalis

(Emery, 1892)

3101FCA1-FF21-5B43-B13C-90373A682481

##### Notes

(Emery 1892, Santschi 1914, Wheeler 1922, Bolton 1982, Medler 1980)

#### ** Monomorium sp1

1164D155-3421-5F3D-925E-274AF382DA86

##### Notes

New Species - to be described in another journal

New Records: 7, 8, 11

#### ** Monomorium afrum

André, 1884

DA4DE0D5-476E-5E76-A406-FC8AF5EB6B7E

##### Notes

New record for Nigeria

New Records: 1, 2, 6

#### * Monomorium bicolor

Emery, 1877

233D4907-725D-5C9E-87AC-DBD950FC4BA3

##### Notes

(Taylor 1980a, Medler 1980, Bolton 1987)

New Records: 1, 2, 3, 4, 6, 7, 8, 9, 10, 12

#### Monomorium egens

Forel, 1910

DC69BDF7-15A7-5F1C-85FC-B2EBE3F9EB33

##### Notes

(Bolton 1987)

#### * Monomorium exiguum

Forel, 1894

0CC385D9-6142-5917-9B34-876F64298621

##### Notes

(Bolton 1987, Sharaf et al. 2018)

New Records: 1, 2, 5, 8, 9, 10

#### * Monomorium floricola

(Jerdon, 1851)

1A387D4E-3772-5206-9CD4-BF110B278292

##### Notes

(Santschi 1914, Wheeler 1922, Medler 1980, Taylor 1980a, Bolton 1987, Wetterer 2010a)

New Records: 2, 4, 8, 10, 11

#### Monomorium invidium

Bolton, 1987

99168008-4CFE-5559-B7C9-AC55EAF6093A

##### Notes

(Bolton 1987)

#### * Monomorium pharaonis

(Linnaeus, 1758)

E31FACB8-8E8F-534B-BC7A-DD76B5888F00

##### Notes

(Medler 1980, Bolton 1987)

New Records: 5, 10

#### * Monomorium rosae

Santschi, 1920

F7520025-6A49-59FA-A74D-AC7D89A32A47

##### Notes

(Bolton 1987)

New Records: 1, 2, 10

#### ** Monomorium vonatu

Santschi, 1930

8E4ED8DA-D0C8-5450-9752-DFBEFE45BE24

##### Notes

New record for Nigeria

New Records: 2

#### Monomorium vaguum

Bolton, 1987

B65E4429-A3EB-5422-9424-4B2CEB304C97

##### Notes

(Bolton 1987)

#### Myrmicaria fumata

Santschi, 1916

4F11A595-680C-5DD0-ADED-E37932DE4F58

##### Notes

(Santschi 1933)

#### Myrmicaria natalensis eumenoides

(Gerstäcker, 1859)

77048AF8-8435-5634-8F8B-342D318844AE

##### Notes

(Medler 1980)

#### Myrmicaria striata

Stitz, 1911

436BB0F3-F231-5037-8DC7-C707F6D45A8A

##### Notes

(Taylor and Adedoyin 1978, Taylor 1980a, Medler 1980, Ewuim 2007)

#### * Nesomyrmex angulatus

(Mayr, 1862)

658BA1B6-C092-58DB-BF6D-63DECEC4DB48

##### Notes

(Bolton 1982, Hita Garcia et al. 2017)

New Records: 1

#### Pheidole aurivillii kasaiensis

Forel, 1911

511DA189-93EA-5FC5-B2E2-0F35A9160EAD

##### Notes

(Forel 1913, Wheeler 1922)

#### ** Pheidole bequaerti

Forel, 1913

2408B85D-0F38-5A08-B0EC-84EA19DA2383

##### Notes

New record for Nigeria

New Records: 2, 6, 9, 10

#### Pheidole caffra

Emery, 1895

7D7B639A-343E-5366-A50C-4AFEDEF432CE

##### Notes

(Medler 1980)

#### ** Pheidole caffra senilifrons

Wheeler, 1922

9EE8FBB1-9C6B-5A3B-B3C5-50F5A8E22AEB

##### Notes

New record for Nigeria

New Records: 3, 5, 8, 11, 12

#### Pheidole crassinoda

Emery, 1895

2C568794-FF5E-5BB0-A944-ECE021FB644C

##### Notes

(Medler 1980, Taylor 1980a, Ewuim et al. 2011)

#### * Pheidole excellens weissi

Santschi, 1910

6935D2A8-C4E6-526D-8842-9909E2431B44

##### Notes

(Santschi 1914, Wheeler 1922)

New Records: 1, 2, 3, 4, 5, 6, 9

#### Pheidole liengmei

Forel, 1894

2C5AC9F6-21F1-5436-99DA-15CAD9B61BC1

##### Notes

(Medler 1980)

#### * Pheidole megacephala

(Fabricius, 1793)

24453B0D-8BBB-5F1A-8DB8-20FB68A87176

##### Notes

(Adenuga 1975, Taylor and Adedoyin 1978, Medler 1980, Taylor 1980a, Wetterer 2012c)

New Records: 2, 3, 5, 7, 8, 9, 10, 11, 12

#### Pheidole megacephala costauriensis

(Fabricius, 1793)

40446295-EC86-52BF-A345-0FE5B387522B

##### Notes

(Santschi 1914, Wheeler 1922, Braet and Taylor 2008)

#### Pheidole megacephala impressifrons

Wasmann, 1905

82B76C0B-407F-5B71-8CE0-D9D07FB50EEB

##### Notes

(Forel 1913, Wheeler 1922, Prins 1963, Prins 1964)

#### Pheidole megacephala melancholica

Santschi, 1912

17CF477B-7D6A-507B-B480-77FD8954B31E

##### Notes

(Bernard 1953)

#### Pheidole minima

Mayr, 1901

2087F3EF-EE29-5618-9F44-101474FC3845

##### Notes

(Taylor and Adedoyin 1978, Taylor 1980a)

#### Pheidole minima catella

Santschi, 1914

CE6809DB-D5C0-56BC-AD55-BB2060231829

##### Notes

(Santschi 1914, Wheeler 1922)

#### Pheidole nigeriensis

Santschi, 1914

322849A3-3CF0-52FF-9E2B-BF1901AFC6A4

##### Notes

(Santschi 1914, Wheeler 1922)

#### Pheidole semidea

Fischer et al., 2012

5368B546-93F8-5098-BDD2-6B6D7CF50AC3

##### Notes

(Fischer et al. 2012)

#### * Pheidole speculifera

Emery, 1877

BDE05B3D-E0DB-54A6-9C4D-FF883C998746

##### Notes

(Medler 1980, Taylor 1980a)

New Records: 1, 5

#### Pristomyrmex orbiceps

(Santschi, 1914)

1F1B0AEA-4255-51F2-90EE-CF4A51A8094F

##### Notes

(Bolton 1981a, Wang 2003)

#### Solenopsis geminata

(Fabricius, 1804)

DFBE36FA-8965-5C4E-B736-DB5C5477400E

##### Notes

(Bolton 1973a, Medler 1980, Taylor 1980a, Wetterer 2011a)

#### ** Solenopsis globularia

(Smith, 1858)

D901E97D-F501-5744-98F5-9547DA1D08ED

##### Notes

New record for Nigeria

New Records: 3, 7, 8, 9, 10, 11

#### Solenopsis orbuloides

André, 1890

F4F0F15E-91E3-50C9-BB11-90C68F968648

##### Notes

(Santschi 1914, Wheeler 1922)

#### Strumigenys cacaoensis

Bolton, 1971

6FD45AB4-1CEA-5465-9025-876C8E0E9F3F

##### Notes

(Taylor 1979, Medler 1980, Bolton 1983, Bolton 2000)

#### ** Strumigenys exunca

(Bolton, 2000)

17005806-EACB-542A-BBDA-60BCED026A27

##### Notes

New record for Nigeria

New Records: 2

#### Strumigenys hastyla

Bolton, 1983

641A463C-2DD7-5813-AE59-A8707E843E09

##### Notes

(Bolton 1983)

#### Strumigenys laticeps

(Brown, 1962)

358364ED-65A0-583B-B956-4ABD93F88912

##### Notes

(Brown 1962, Bolton 1972, Medler 1980, Bolton 1983, Bolton 2000)

#### Strumigenys ludovici

Forel, 1904

D5BABC56-C02B-5A76-A25A-0AA079244375

##### Notes

(Taylor 1979, Bolton 1983, Bolton 2000)

#### Strumigenys lujae

Forel, 1902

5CCDAA6D-CD4C-5E6A-B505-9D7D34E727D7

##### Notes

(Medler 1980, Bolton 1983)

#### Strumigenys malaplax

(Bolton, 1983)

EA3EEDA9-343E-5547-AA33-E275C9420BC6

##### Notes

(Bolton 1983)

#### Strumigenys maynei

Forel, 1916

3541EC98-8469-5F78-9A90-1A8606C67296

##### Notes

(Taylor 1979, Medler 1980, Bolton 1983)

#### Strumigenys ninda

(Bolton, 1983)

D92D1C49-90C3-548D-A984-C082410D9FDB

##### Notes

(Bolton 1983, Bolton 2000)

#### Strumigenys pallestes

Bolton, 1971

CED7CB1D-9BFB-56C9-B78B-1A62FF3B8067

##### Notes

(Taylor 1979, Medler 1980, Bolton 1983)

#### * Strumigenys petiolata

Bernard, 1953

4DB662B0-3B39-5A32-83FF-FE8DA8726876

##### Notes

(Bolton 1983, Bolton 2000)

New Records: 2, 8

#### Strumigenys rogeri

Emery, 1890

04700C15-49CD-5F33-A8E5-27A5799ECC01

##### Notes

(Taylor 1979, Bolton 1983, Wetterer 2012b)

#### * Strumigenys rufobrunea

Santschi, 1914

E1700FC6-4E64-5C22-B31D-B4463145C173

##### Notes

(Santschi 1914, Wheeler 1922, Brown 1954, Taylor 1979, Medler 1980, Bolton 1983)

New Records: 1

#### Strumigenys scotti

Forel, 1912

CF0DF045-315D-589C-B5DD-58EA80C61B37

##### Notes

(Taylor 1979, Medler 1980)

#### Strumigenys simoni

Emery, 1895

A5AA3BB2-F353-5224-8EFB-B4B1383FD973

##### Notes

(Santschi 1914, Wheeler 1922, Brown 1952, Bolton 1983, Bolton 2000)

#### Strumigenys vazerka

Bolton, 1983

4F11923B-46F6-586A-B254-A46727284A30

##### Notes

(Bolton 1983)

#### Syllophopsis cryptobia

Santschi, 1921

41E01D3B-C9CF-5A02-88C2-9C5CCE37D69C

##### Notes

(Bolton 1987, Sharaf and Aldawood 2013)

#### Terataner elegans

Bernard, 1953

014EC01C-7959-5710-961B-5ECCAE5C1911

##### Notes

(Bolton 1981a)

#### Terataner luteus

(Emery, 1899)

74D3E7F8-EB45-54B2-B74B-42871B3799F3

##### Notes

(Medler 1980)

#### Terataner piceus

Menozzi, 1942

6380032E-E0C1-5EE7-A708-94474FD60A93

##### Notes

(Bolton 1981a)

#### * Tetramorium aculeatum

(Mayr, 1866)

93DEAA17-8D7E-598F-AE07-E825A99DF45D

##### Notes

(Wheeler 1922, Menozzi 1926, Taylor and Adedoyin 1978, Taylor 1979, Bolton 1980, Medler 1980)

New Records: 5

#### Tetramorium africanum

(Mayr, 1866)

F32093CF-6110-509A-81DE-24462EB0B05D

##### Notes

(Bolton 1980)

#### * Tetramorium angulinode

Santschi, 1910

AF96405F-008C-5782-9073-7BE8E340CD3B

##### Notes

(Bolton 1980)

New Records: 1, 2, 6, 8

#### Tetramorium asetyum

Bolton, 1980

24E0EBED-288C-54B7-93D0-9BC3E9875343

##### Notes

(Bolton 1980)

#### * Tetramorium ataxium

Bolton, 1980

41E01E62-4353-503E-AE3E-AB3245325DA8

##### Notes

(Bolton 1980)

New Records: 8

#### Tetramorium bellicosum

Bolton, 1980

9F2C0D75-B0BB-504D-B867-C62107EB3ED5

##### Notes

(Bolton 1980)

#### ** Tetramorium bicarinatum

(Nylander, 1846)

D2DE455B-7E87-51DE-9FD7-942F42C23FED

##### Notes

New record for Nigeria

New Records: 3

#### Tetramorium boltoni

Hita Garcia et al., 2010

5C9CE663-BAED-5525-B096-0C8232F891E7

##### Notes

(Hita Garcia et al. 2010, Hita Garcia and Fisher 2014a)

#### Tetramorium brevispinosum

(Stitz, 1910)

436D690E-4947-5736-82C2-A26D24813C8F

##### Notes

(Bolton 1976)

#### Tetramorium caldarium

(Roger, 1857)

1C042A78-76FA-580B-B032-0DB14F4FEF1D

##### Notes

(Bolton 1980, Wetterer and Hita Garcia 2015)

#### * Tetramorium calinum

Bolton, 1980

7CCC6AD3-8568-5834-834F-98A8091DA201

##### Notes

(Bolton 1980)

New Records: 1

#### ** Tetramorium cristatum

Stitz, 1910

6487D210-1D2E-5759-AB8C-1D0724A09AFE

##### Notes

New record for Nigeria

New Records: 1

#### Tetramorium critchleyi

(Bolton, 1976)

68068C05-0ABA-5737-B7CC-48AE6391D9B0

##### Notes

(Bolton 1976, Bolton 1986)

#### Tetramorium delagoense

Forel, 1894

9BC27DEC-9188-5C66-BFBB-570DB76B189B

##### Notes

(Bolton 1980)

#### Tetramorium dumezi

Menozzi, 1942

35A82F27-0FBC-5D3F-AA9F-1C790B166C53

##### Notes

(Bolton 1980)

#### Tetramorium dysderke

Bolton, 1980

984C5EA9-9B45-5283-B4C2-9CC91F0EC37E

##### Notes

(Bolton 1980)

#### ** Tetramorium edouardi

Forel, 1894

366AA5C9-2A2B-5561-9C22-CB984600A95D

##### Notes

New record for Nigeria

New Records: 1, 6

#### * Tetramorium eminii

(Forel, 1894)

4C0D834F-2F01-592B-B403-05076E06F895

##### Notes

(Bolton 1976)

New Records: 2

#### ** Tetramorium ericae

Arnold, 1917

522A88E8-E892-5CCC-B9B6-866D1DFD06C3

##### Notes

New record for Nigeria

New Records: 2

#### Tetramorium flavithorax

(Santschi, 1914)

287D7E54-3954-5E84-A3BB-4F41A3CE5A10

##### Notes

(Bolton 1980, Hita Garcia et al. 2010)

#### ** Tetramorium furtivum

(Arnold, 1956)

03AB8434-181B-5A9F-99C8-7E201652FF0B

##### Notes

New record for Nigeria

New Records: 8, 10

#### Tetramorium granulatum

Arnold, 1956

CB8C357B-8EDF-5BB1-BF3B-2070F24C741C

##### Notes

(Bolton 1980, Taylor et al. 2018)

#### Tetramorium guineense

(Bernard, 1953)

0FB423A4-B5F3-5BA0-B1C8-5E682446CB14

##### Notes

(Bolton 1980, Hita Garcia et al. 2010, Hita Garcia and Fisher 2014a)

#### Tetramorium intonsum

Bolton, 1980

32D610AE-C8BB-53AE-A2AA-78C7EFA2F1E8

##### Notes

(Bolton 1980)

#### Tetramorium jugatum

Bolton, 1980

FA19257D-CBC1-5222-A164-B946288212EE

##### Notes

(Bolton 1980)

#### Tetramorium khyarum

Bolton, 1980

1861E730-3783-56EC-825F-B48F8E37FE36

##### Notes

(Bolton 1980, Sharaf and Al-Zailaie 2006)

#### ** Tetramorium lanuginosum

Mayr, 1870

DF3E3D25-C4C1-5860-94B3-08591E89D185

##### Notes

New record for Nigeria

New Records: 2, 7

#### * Tetramorium legone

Bolton, 1980

9158DC0D-5316-5912-BE79-84DFE70D8536

##### Notes

(Bolton 1980)

New Records: 1

#### Tetramorium longicorne

Forel, 1907

1FE24877-3BC5-5662-9554-9B66AA0A0CD1

##### Notes

(Bolton 1980)

#### Tetramorium lucayanum

Wheeler, 1905

AC8604E3-A1B1-5BB6-BBF0-36056144DD10

##### Notes

(Santschi 1914, Wheeler 1922, Brown 1964, Taylor 1980a, Bolton 1980, Wetterer 2011b)

#### Tetramorium minimum

(Bolton, 1976)

0CB38853-1BF2-512D-8D85-C8779D57C0B1

##### Notes

(Bolton 1976)

#### Tetramorium opacum

(Forel, 1909)

807A9547-5F93-5694-B6B4-6B504EAE54A5

##### Notes

(Medler 1980)

#### * Tetramorium quadridentatum

Stitz, 1910

E12B1FD4-2631-53C3-B98B-4A920122823E

##### Notes

(Bolton 1980)

New Records: 10

#### Tetramorium raptor

Hita Garcia & Fisher, 2014

BBB76047-7265-5CE3-BDF7-CBB959514CCA

##### Notes

(Medler 1980, Taylor 1980a, Hita Garcia and Fisher 2014b; as decem)

#### Tetramorium rubrum

Hita Garcia et al., 2010

36F5981A-6799-54EE-96FF-84F5C0EF5E6A

##### Notes

(Hita Garcia et al. 2010)

#### * Tetramorium sericeiventre

Emery, 1877

8E8DCC89-FBBD-5D57-9056-D5FEAF74A9B0

##### Notes

(Santschi 1914, Wheeler 1922, Bolton 1980, Medler 1980, Taylor 1980a)

New Records: 1, 2, 3, 6, 7, 8, 10

#### * Tetramorium simillimum

(Smith, 1851)

A698C094-3538-56F9-BD04-E88364765F0D

##### Notes

(Santschi 1914, Wheeler 1922, Bolton 1980)

New Records: 1, 2, 3, 6, 7, 8, 10, 11

#### * Tetramorium uelense

Santschi, 1923

D4C63EB4-5BDE-5039-BF9D-26E6B291F41E

##### Notes

(Longhurst et al. 1979, Hita Garcia and Fisher 2014b)

New Records: 1

#### Tetramorium wadje

Bolton, 1980

4C450D61-AA70-509D-9930-43924F126883

##### Notes

(Bolton 1980)

#### ** Tetramorium xuthum

Bolton, 1980

B4AB16A0-80EF-54AD-9B38-D2A276F90552

##### Notes

New record for Nigeria

New Records: 6

#### * Tetramorium zapyrum

Bolton, 1980

1DD6A333-9728-5951-A6CF-E4823AA1E39F

##### Notes

(Bolton 1980)

New Records: 1, 6, 8

#### Trichomyrmex abyssinicus

(Forel, 1894)

794522E2-EFDC-5413-B140-DDB3CACFC928

##### Notes

(Bolton 1987, Radchenko 1997, Rigato 2002, Sharaf et al. 2016)

#### ** Trichomyrmex destructor

(Jerdon, 1851)

2D6CAD2F-BFBF-5FA5-AB8C-BA9FC1067854

##### Notes

New record for Nigeria

New Records: 2, 9, 11

#### ** Trichomyrmex mayri

(Forel, 1902)

4E5A44B8-E3EC-5163-92C9-9B7E25D0D8A7

##### Notes

New record for Nigeria

New Records: 8

#### Ponerinae

5892576E-72B4-5869-A10D-85A2B019BF62

#### Anochetus africanus

(Mayr, 1865)

48114EEE-1719-5A0E-BA08-8FDB304B6064

##### Notes

(Taylor 1976, Medler 1980)

#### Anochetus bequaerti

Forel, 1913

70A34EC6-82CE-5376-9BD8-A09C167CE0F7

##### Notes

(Taylor 1976, Medler 1980)

#### Anochetus fuliginosus

Arnold, 1948

B2CC64DB-CAB8-5383-911D-1851C9644615

##### Notes

(Brown 1978)

#### Anochetus katonae

Forel, 1907

4D393590-7A15-583E-B8FA-4126357E6858

##### Notes

(Santschi 1914, Wheeler 1922, Medler 1980)

#### Anochetus pellucidus

Emery, 1902

429DA267-E005-5918-99C4-61FAC6DF8526

##### Notes

(Taylor 1976, Brown 1978, Medler 1980)

#### Anochetus punctaticeps

Mayr, 1901

68CD6F42-E890-5233-AD87-7DD4D8241065

##### Notes

(Taylor 1976, Medler 1980)

#### Anochetus siphneus

Brown, 1978

E18E1914-D77E-570D-A1F4-4FBC1EE9C2CD

##### Notes

(Brown 1978)

#### Anochetus talpa

Forel, 1901

F53B9DBB-4B23-553C-AEAC-3E161E651697

##### Notes

(Santschi 1914, Wheeler 1922, Medler 1980)

#### Asphinctopone silvestrii

Santschi, 1914

875C595A-3069-5DDF-88E1-B797CF7A8D2D

##### Notes

(Santschi 1914, Wheeler 1922, Weber 1949, Lévieux 1972, Medler 1980, Bolton and Fisher 2008b)

#### Bothroponera ryderae

Joma & Mackay, 2017

4855B53F-7B9A-5238-B817-EC3527E4695E

##### Notes

(Joma and MacKay 2017)

#### * Bothroponera silvestrii

(Santschi, 1914)

A42294B8-80D7-5267-B249-6DB6EAAF0DEC

##### Notes

(Taylor 1976, Joma and MacKay 2017)

New Records: 8

#### Bothroponera soror

(Emery, 1899)

E131A04D-3A0F-55F3-BC18-99A9B4868EBD

##### Notes

(Medler 1980)

#### * Brachyponera sennaarensis

(Mayr, 1862)

3F873D55-FF4E-53A5-9517-2639191FC642

##### Notes

(Santschi 1914, Wheeler 1922, Prins 1963, Prins 1964, Taylor 1976, Medler 1980, Rafinejad et al. 2009, Wetterer 2013a)

New Records: 2, 3, 5, 7, 8, 10

#### Centromyrmex sellaris

Mayr, 1896

7F374EAE-CEA6-5C67-B09A-7CB2781B25FA

##### Notes

(Taylor 1976, Bolton and Fisher 2008a)

#### Euponera brunoi

(Forel, 1913)

110D573E-AF67-5692-AA3D-05591CEA0444

##### Notes

(Santschi 1914, Wheeler 1922, Brown 1963, Taylor 1976, Medler 1980)

#### Euponera sjostedti

(Mayr, 1896)

C4ECA32B-2BAE-5847-90DB-8AE1903496A9

##### Notes

(Taylor 1976, Medler 1980)

#### Hypoponera angustata

(Santschi, 1914)

883102A5-7BD3-5F8C-AF16-CE72D35E1CB8

##### Notes

(Bolton and Fisher 2011)

#### Hypoponera camerunensis

(Santschi, 1914)

14AD7B3E-1C84-53EE-8EBB-4948AF87D156

##### Notes

(Taylor 1976, Medler 1980)

#### Hypoponera coeca

(Santschi, 1914)

5C2183F8-ACB4-5C66-86DC-2B64C4096249

##### Notes

(Bolton and Fisher 2011)

#### Hypoponera dulcis

(Forel, 1907)

CBAC82C4-A03A-546A-B680-258A3EF0CF95

##### Notes

(Bolton and Fisher 2011)

#### Hypoponera inaudax

(Santschi, 1919)

65E13800-5C37-595D-A0FA-10BAB3820A22

##### Notes

(Bolton and Fisher 2011)

#### Hypoponera lea

(Santschi, 1937)

C8BF195F-7980-54C7-84F3-2C276E0CE113

##### Notes

(Taylor 1976)

#### Hypoponera lepida

Bolton & Fisher, 2011

819EA39A-9E39-55CF-B195-8B0C29AFF41F

##### Notes

(Bolton and Fisher 2011)

#### Hypoponera punctatissima

(Roger, 1859)

9688893E-28D2-5534-9AEF-56F4F2F6DFDF

##### Notes

(Santschi 1914, Wheeler 1922, Bernard 1953, Taylor 1976, Medler 1980, Delabie and Blard 2002, Nwosu and Lawal 2010, Bolton and Fisher 2011)

#### ** Leptogenys conradti

Forel, 1913

48FAD9BA-04FF-5790-B5D2-81DDDD80131B

##### Notes

New record for Nigeria

New Records: 6

#### Leptogenys elegans

Bolton, 1975

33367B65-E73C-5DBF-AB71-09ABF4F9CFE6

##### Notes

(Bolton 1975b, Taylor 1976, Medler 1980)

#### Leptogenys khaura

Bolton, 1975

39DA4090-B65C-5B49-80DB-5FEDA0BBBFAF

##### Notes

(Bolton 1975b, Medler 1980)

#### ** Leptogenys longiceps

Santschi, 1914

1F73E1BD-D730-51BF-9A19-30FC64DD5780

##### Notes

New record for Nigeria

New Records: 1, 2

#### Leptogenys stygia

Bolton, 1975

2995D0A6-3BD0-53FF-BF97-49510972A991

##### Notes

(Bolton 1975b, Taylor 1976, Medler 1980)

#### Loboponera politula

Bolton & Brown, 2002

5472DBFC-7736-5576-A5B0-55ABEE554AF7

##### Notes

(Bolton and Brown Jr 2002)

#### Loboponera vigilans

Bolton & Brown, 2002

8D04B268-FCAC-5D88-A022-F2B8D83747D7

##### Notes

(Bolton and Brown Jr 2002)

#### Megaponera analis

(Latreille, 1802)

9147AA35-834E-5768-8F63-AE112A05C8F2

##### Notes

(Wheeler 1922, Prins 1963, Prins 1964, Medler 1980, Ewuim 2007)

#### Mesoponera ambigua

(André, 1890)

8B26354E-E781-5C45-906A-E424DA8F8DAE

##### Notes

(Taylor 1976, Medler 1980)

#### * Mesoponera caffraria

(Smith, 1858)

F003393D-94E1-572C-9318-F1CF5C51BB3C

##### Notes

(Taylor 1976, Medler 1980)

New Records: 1, 5, 10

#### * Odontomachus troglodytes

Santschi, 1914

C024ABEA-0A49-5F29-A6D2-7EACB1C51D51

##### Notes

(Santschi 1914, Wheeler 1922, Prins 1963, Prins 1964, Taylor 1976, Taylor and Adedoyin 1978; as *O.haematodus*, Medler 1980, Ewuim et al. 2011)

New Records: 1, 2, 4, 5, 8, 9, 10, 11, 12

#### * Paltothyreus tarsatus

(Fabricius, 1798)

15B3A760-2C2D-5E87-AAB3-4D02BDD31B5E

##### Notes

(Taylor 1976, Medler 1980, Ewuim et al. 2011)

New Records: 1, 2, 5, 6, 8, 10, 11

#### Parvaponera darwinii africana

(Forel, 1909)

0DAB7827-7F7E-5EE2-BF42-01FB2AC62E26

##### Notes

(Santschi 1914, Wheeler 1922, Santschi 1935)

#### Platythyrea conradti

Emery, 1899

970D920D-CE43-51C0-B5B9-23925B224B27

##### Notes

(Taylor 1976, Medler 1980)

#### Platythyrea frontalis

Emery, 1899

E31AA611-AE77-5694-9377-722B86112EB5

##### Notes

(Medler 1980)

#### Platythyrea lamellosa

(Roger, 1860)

1AC4E571-572C-566B-B54E-BD63DEAB8BE7

##### Notes

(Medler 1980)

#### Platythyrea modesta

Emery, 1899

465DBABA-E857-5EE6-9EA0-1BAD76EFEFA6

##### Notes

(Taylor 1976)

#### Plectroctena cristata

Emery, 1899

5998C136-EFF5-5FDE-9E18-F2005046ED80

##### Notes

(Medler 1980)

#### Plectroctena macgeei

Bolton, 1974

54B3781B-A27D-57B6-8125-F0A99FFB36AA

##### Notes

(Bolton 1974b, Taylor 1976, Medler 1980, Bolton and Brown Jr 2002)

#### Plectroctena minor

Emery, 1892

3BB2517E-4AA1-55A7-B6E8-F1AD6A65E53E

##### Notes

(Bolton 1974b, Taylor 1976, Medler 1980)

#### Psalidomyrmex foveolatus

André, 1890

4B1EA43F-E2FD-5E00-ABC2-BF19E1347D31

##### Notes

(Bolton 1975a, Taylor 1976, Medler 1980, Bolton and Brown Jr 2002, Taylor et al. 2018)

#### Proceratiinae

D06C92F0-CAC4-50AD-BBDB-E769907A920B

#### Discothyrea oculata

Emery, 1901

E6286206-235E-514C-A9AE-05435C9B6596

##### Notes

(Hita-Garcia et al. 2019)

#### Pseudomyrmecinae

FD0F62F5-D6BC-598F-8C14-D359F7B349F2

#### Tetraponera aethiops

Smith, 1877

FE268C44-5CD2-5363-960A-339F384097C6

##### Notes

(Forel 1913, Wheeler 1922, Medler 1980)

#### Tetraponera anthracina

(Santschi, 1910)

26694D26-2A97-52E9-BBD2-7CF91BDDBF85

##### Notes

(Santschi 1937b, Taylor 1976, Taylor and Adedoyin 1978, Medler 1980)

#### Tetraponera latifrons

(Emery, 1912)

CAA0374C-C358-5A5D-B57C-EBAD3DA5BB12

##### Notes

(Medler 1980)

#### * Tetraponera mocquerysi

(André, 1890)

E34829E7-2C99-5870-ADD5-ADED4C78B167

##### Notes

(Santschi 1914, Wheeler 1922, Baroni Urbani 1977a)

New Records: 1

#### Tetraponera ophthalmica

(Emery, 1912)

5B5965F4-E037-5BCB-8BC1-B463C36A1781

##### Notes

(Taylor 1976)

## Analysis

A total of 106 native and exotic ants were recorded in this study, out of these, 28 species are new records for Nigeria and 28 are unidentified to species level. Ten exotic and potentially invasive species were also recorded. Four exotic species were listed in previous publications and were also encountered in this study: *Paratrechinalongicornis*, *Monomoriumfloricola*, *Monomoriumpharaonis* and *Tapinomamelanocephalum*; six species were added from the present study: *Nylanderiabourbonica*, *Solenopsisglobularia*, *Tetramoriumbicarinatum*, *Tetramoriumlanuginosum*, *Trichomyrmexdestructor* and *Trichomyrmexmayri*. *Solenopsisgeminata* was also listed in the previous study, but was not found in the present study. This brings the update of exotic species to 11 (Table 1). Three tramp species: *Cardiocondylaemeryi*, *Pheidolemegacephala* and *Tetramoriumsimillimum*, that are native to Nigeria are also recorded (Table 1). The 132 reviewed journals yielded 288 species/subspecies and, in addition to ants found in this study (28), an updated record of Nigeria ants is now 316, all from 10 subfamilies and 64 genera.

### Updated list of Nigerian ants

An additional 28 collected morphospecies were not included in the Nigerian checklist because a proper identification could not be achieved due to the current challenges in the taxonomy of these genera or because they were represented only by sexual forms and only generic identification could be assessed. These morphospecies belong to: *Cardiocondyla* (1), *Carebara* (4), *Cataulacus* (3), *Crematogaster* (6), *Dorylus* (4), *Lepisiota* (1), *Myrmicaria* (1), *Pheidole* (2), *Plagiolepis* (1), *Tapinoma* (2), *Solenopsis* (1), *Strumigenys* (1) and *Tetramorium* (1).

A different case is the species listed as *Monomorium* sp1, as its status as a species is unknown to science, but it is not described in this article.

### Species deleted from the list

*Aenictusrotundatus* Mayr, 1901: *Aenictus* has been recently revised by one of the authors (Gómez 2022) and the distribution of this species seems to be restricted to eastern and southern Africa. Its sibling species *Aenictusguineensis* is distributed throughout western Africa, from Senegal to Nigeria (samples from Ibadan and Mokwa examined). Therefore, Nigerian records of *A.rotundatus* are here transferred to *Aenictusguineensis*.

*Anochetusjonesi* Arnold, 1926: described from South Africa. Its listing came from Taylor (1979) and was, therefore, added to the 1980 catalogue (Medler 1980). Taylor (1979) did not list this species from Nigeria in his web publication - The Ants of Africa - it is thus removed from the Nigerian list.

*Camponotusbarbarossamicipsa* Wheeler, 1922 was cited in Taylor (1978), but the identification changed to *Camponotusbarbarossa* Emery, 1920 by Taylor in his site, Ants of Africa (Taylor 2021).

*Camponotusforaminosusdorsalis* Santschi, 1926: subspecies described from Tanzania, was listed by Taylor (1978) and Taylor and Adedoyin (1978) and then added to the 1980 catalogue. Taylor (1978) gave no particular reasons to identify the Nigerian material within this subspecies. It has not been listed anywhere apart from the original description by Santschi (1926). As a rule of thumb, East African and West African ant faunas tend to be distinct. It is, therefore, better to follow the cautious approach of naming the Nigerian material as *C.foraminosus* sensu lato until a thorough revision involving all subspecies suggests otherwise.

*Camponotusrufoglaucus* Jerdon, 1851: listed in a catalogue (Medler 1980), but this reference could not be traced to any previous record. It is an Asian species with five subspecies listed from the Afrotropical Region. All the records for the species and subspecies are from eastern and southern Africa. It was, therefore, not included in the list of Nigerian species.

*Cardiocondylazoserka* Bolton, 1982: The current status of this species remains unclear, since Heinze (2019) discovered that the type material from Nigeria are males and not queens as previously thought and maybe belong to a previously-described species. We have opted for listing this species until its taxonomical status is cleared.

*Messorbarbarus* Linnaeus, 1767: listed in Medler (1980), it is a European species and the record could not be traced to any previous source. Most definitely a misidentification of *Messorgalla* which is quite similar and widespread in the Afrotropical Region north of the Equator, as well as Kenya and Tanzania.

*Odontomachushaematodus* Linnaeus, 1758: South American species listed by Santschi (1914) from Ibadan, Lagos and Olokomeji and subsequently recorded by other authors (Wheeler 1922, Prins 1963, Prins 1964). It is clearly a misidentification of the common *Odontomachustroglodytes* and the record has been transferred to this species.

*Technomyrmexalbipes* Smith, 1861: tramp species distributed worldwide. Its presence in Nigeria was first recorded by Taylor (1978) and it was added to the Medler (1980) catalogue. Taylor in his website ‘The Ants of Africa’ did not record this species from Nigeria and the only other specimen under this name collected in Ghana was re-identified as *T.andrei* “which seems to be quite common in Nigeria and possibly Ghana” (Taylor 2021). So, the identification was transferred to *T.andrei*.

*Tetramoriumdecem* Forel, 1913: listed in Medler (1980) and by Taylor (1980a). The group was subsequently revised by Hita Garcia and Fisher (2014b), stating this species as eastern and southern African. Taylor in his website “The Ants of Africa'' acknowledges this distribution and transferred his samples from Nigeria to *T.raptor*, so this study follows the same pattern.

*Tetraponerapenzigi* Mayr, 1907: listed in Medler (1980) with no other reference. It seems to have an eastern and southern African distribution and antmaps.org lists it as “dubious”, based on personal communication by Dr. Phil Ward. So, this is not included until new data are available.

### Notes to the list additions

*Camponotusschoutedeni*: widely distributed species, has been reported from South Africa to Côte d’Ivoire.

*Cardiocondylasekhemka*: this is the second record of this species worlwide. Until this present study, this species was known only from its type locality in Tumu, Ghana (Bolton 1982). Four workers and one queen were collected in pitfall traps at Badagry.

*Cardiocondylaweserka*: known from Cameroon, was represented in a collection from Badagry by one queen that was assigned to this species, based on the morphology of its worker caste. The queen caste is currently undescribed.

*Cardiocondylayoruba*: endemic species described in Rigato (2002), based on specimens from Ibadan. It was found at seven different sites, showing that it could be more common than estimated in the oryginal description.

*Crematogasterlamottei*: species listed from Guinea (type loc.) and Central African Republic by Bernard (1953). This species belongs to the *kneri* species group, formerly subgenusSphaerocrema. Morphometric analysis by one of the authors (KG) reveals that it is almost identical to the much more common and widespread *C.striatula*, also found in the samples collected in this study. The main differences between these species are the longer propodeal spines in *C.lamottei*, all the other characteristics being widely variable and overlapping. The real status of this species should be determined in a generic revision.

*Dorylusbraunsi*: the identity of *Dorylus* species is unreliable due to lack of a revision of the genus to date. So, identity of *D.braunsi* is based on comparison with types from antweb.org and should, therefore, be taken with caution (Fisher 2023).

*Lepisiotaambigua*: known from Democratic Republic of Congo, but it seems to be distributed throughout West Africa as it was collected in two countries by two of the authors, Kiko Gómez in Senegal and Natasha Mothapo in University of Ghana and also at Kwame Nkrumah Memorial Park in Ghana (unpublished).

*Leptogenysconradti* and *Leptogenyslongiceps*: both species have West African distribution and their presence is confirmed here.

*Monomoriumafrum*: widely distributed species throughout tropical Africa, from Senegal to Côte d’Ivoire. Collection in this study is the first record for Nigeria.

*Monomoriumvonatu*: known from Burkina Faso, Ghana and Senegal, the records in this study extend its distribution to Central Africa.

*Monomorium* sp1: This is a new species being described in another publication (KG, in preparation). Its distribution ranges from Senegal to Nigeria.

*Nylanderiabourbonica*: Asian introduced species known in Africa only from unpublished records in Tanzania.

*Nylanderiaumbella*: known from a few locations (Cameroon, Gabon and Uganda), our records extending its range to the northwest: Senegal (KG leg.) and Nigeria.

*Pheidolebequaerti*: new species to Nigeria; previously recorded from Benin, Senegal and DRC.

*Pheidolecaffrasenilifrons*: known from Central Africa (DRC, Congo, Gabon, Cameroon, Benin and Togo), new to Nigeria.

*Solenopsisglobularia*: known from Côte d’Ivoire and Senegal; it is an invasive ant species spreading throughout West Africa. It seems to be well established in Lagos State and is likely spreading to other areas of the country.

*Strumigenysexunca*: first Nigerian record for this West African species, previously recorded from Cameroon, Ghana and Côte d’Ivoire.

*Tapinolepispernix*: known from Sudan (type loc.), Benin and Senegal. This genus also needs a revision. The species was assigned to this species by comparing it with the photographs from antweb.org (Fisher 2023).

*Tetramoriumbicarinatum*: exotic species with worldwide distribution. It has been collected from Guinea and Ghana in West Africa and the DRC in Central Africa.

*Tetramoriumcristatum*, *Tetramoriumedouardi*, *Tetramoriumfurtivum*: West African and Congo Basin species, recorded in Nigeria for the first time.

*Tetramoriumericae*: the identification of this species must be taken with caution. Its measurements fit the description and it is virtually identical to the type images in Antweb, but its distribution seems to be restricted to southern and eastern Africa (Fisher 2023).

*Tetramoriumlanuginosum*: exotic species known in the Afrotropical Region only from Ghana and Sierra Leone and labelled as “dubious” in the Antmaps site (Guénard et al. 2017).

*Tetramoriumxuthum*: first world record apart from the type series from Ghana.

*Trichomyrmexdestructor*: first record of this invasive species in Nigeria. It has been recorded extensively in West and Central Africa.

*Trichomyrmexmayri*: first record for Nigeria. Its presence had earlier been recorded in Sahelian Mali, Niger, Senegal and Sudan.

## Discussion

The study aimed to ascertain the prevalence of alien/exotic/potential invasive ant species in Nigeria and to reassess the checklist of species first compiled in the 1970s. A total of 28 new ant species were added in the updated checklist and the number of exotic species recorded has increased to 11. Further, eleven species were excluded from the current checklist largely due to prior misidentifications. These additions and removals resulted in a total of three hundred and sixteen (316) ant species in the checklist of ants of Nigeria. Increased sampling and identification efforts are yielding better knowledge of ant fauna composition in the region. This important finding, particularly in relation to the exotic species showed that there has been an increase in the number of invasive species which may have significant negative impacts on the native ant fauna. The invasive ant fauna should be monitored in future studies and its real distribution and impact should be assessed.

In recent years, research has indicated the growing number of invasive species hotspots and the association of urbanisation with these hot spots. Most invasive ant species occur in urbanised environments, which may facilitate the spread of propagules of invasive species into natural environments (Van Ham et al. 2013). Without the ecological studies to ascertain the impact of the presence of these invasive species on native ant fauna, a final assessment of the species presence in the country cannot be made. Future work should focus on the ecological interactions amongst these species as basic studies are missing in the region and on a large part of the African continent.

## Figures and Tables

**Table 1.** Current list of 14 tramp/exotic/species in Nigeria. Species with asterisk (*) indicate a new addition.

**Species**	**Purported (Putative) Native range**	**Status**	**References**
*Cardiocondylaemeryi*	Sub-Saharan Africa	Native/Tramp	Seifert (2003), Wetterer (2012a)
*Monomoriumfloricola*	Tropical Asia	Exotic/Potential invasive	Abbott et al. (2006), Wetterer (2010a)
*Monomoriumpharaonis*	Tropical Asia	Exotic/Potential invasive	Wetterer (2010c)
*Nylanderiabourbonica	Southern and South-East Asia	Exotic/Potential invasive	Williams and Lucky (2020)
*Paratrechinalongicornis*	Indo-Pacific (South East Asia & Melanesia)	Exotic/Potential invasive	Wetterer (2008)
*Pheidolemegacephala*	Sub-Saharan Africa	Native/Tramp	Wetterer (2012b)
*Solenopsisglobularia	Neotropical & Nearctic	Exotic/Potential invasive	Wetterer (2019)
*Solenopsisgeminata*	New World (South and Central America)	Exotic/Potential invasive	Wetterer (2011a)
*Tapinomamelanocephalum*	Indo-Pacific	Exotic/Potential invasive	Wetterer (2009a)
*Tetramoriumbicarinatum	Indo-Pacific	Exotic/Potential invasive	Wetterer (2009c)
*Tetramoriumlanuginosum	India, East Asia, Northern Australia and Western Oceania	Exotic/Potential invasive	Wetterer (2010b)
*Tetramoriumsimillimum*	Afrotropical region	Native/Tramp	Bolton (1980)
*Trichomyrmexdestructor	North Africa, Middle East and South Asia	Exotic/Potential invasive	Wetterer (2009b)
*Trichomyrmexmayri	Indian Subcontinent	Exotic/Potential invasive	Bolton (1987), Borowiec and Salata (2019)

**Table 2.** Description of collecting sites in Lagos State.

**Locality code**	**Locality name**	**State**	**Habitat**	**Coordinates**	**Altitude (m)**
1	Agbowa	Lagos	Agricultural Cassava farm, grassland	6.6608, 3.72519	10 m
2	Badagry	Lagos	Agricultural Cassava farm, grassland. Dominant Tree- *Cocosnucifera*	6.42682, 2.83925	10 m
3	Alimosho (Ikotun)	Lagos	Urban, Residential Area	6.57519, 3.27331	10 m
4	Faculty of Science, University of Lagos (UNILAG)	Lagos	Garden	6.51662, 3.39922	10 m
5	IITA Ibadan	Ibadan	Reserve/Urban	7.50121, 3.908	200 m
6	Ilogbo Eremi	Lagos	Agricultural Cassava farm, grassland.	6.50018, 2.97196	
7	Kosofe (Ketu)	Lagos	Urban, Residential Area	6.59126, 3.40238	10 m
8	LUFASI Nature park	Lagos	Reserve Area, Tropical grassland. Dominant Tree- Palm trree	6.46805, 3.65462	10 m
9	Mushin (Ilupeju)	Lagos	Urban, Residential Area	6.54909, 3.35996	10 m
10	NCF/LCC (Lekki Conservation Center)	Lagos	Reserve Area, Tropical grassland	6.43219, 3.53552	10 m
11	Omu Resort	Lagos	Reserve Area, Tropical grassland	6.49011, 3.75319	10 m
12	Victoria Garden City (VGC)	Lagos	Urban Residential Area	6.46048, 3.54931	10 m
